# Prevalence and Seasonal Patterns of 16 Common Viral Respiratory Pathogens during the COVID-19 Pandemic in Gauteng Province, South Africa, 2020–2021

**DOI:** 10.3390/v16081325

**Published:** 2024-08-19

**Authors:** Bhaveshan Reddy, Andiswa Simane, Hloniphile Mthiyane, Bonolo Mashishi, Nonhlanhla Mbenenge, Florette K. Treurnicht

**Affiliations:** 1 Division of Virology, School of Pathology, Faculty of Health Sciences, University of the Witwatersrand, Johannesburg 2193, South Africa; 2 National Health Laboratory Service, Johannesburg 2192, South Africa

**Keywords:** COVID-19, respiratory viruses, SARS-CoV-2, prevalence, seasonal respiratory viruses

## Abstract

Coronavirus disease 2019 (COVID-19) is caused by the severe acute respiratory syndrome coronavirus 2 (SARS-CoV-2). The COVID-19 pandemic resulted in widespread morbidity and mortality, but generally, the diagnosis of other respiratory viruses was limited. This study aimed to assess the prevalence of other respiratory viruses during the 2020/2021 pandemic among patients of all ages who accessed care at public healthcare facilities in Gauteng Province, South Africa. Laboratory diagnosis for respiratory viruses, with or without SARS-CoV-2, was conducted via multiplex real-time polymerase chain reactions using respiratory specimens. A total of 1776 patients were included from 1 April 2020 to 31 March 2021, of which 766 (43.1%) were positive for respiratory viruses other than SARS-CoV-2. RV (368/1776; 20.7%) was the most prevalent, followed by RSV (304/1776; 17.1%), AdV (112/1776; 6.3%) and EV (105/1776; 5.9%). hCoV-OC43 (39/1776; 2.2%) was the most prevalent common coronavirus. SARS-CoV-2 co-infections were detected in 4.8% (24/500) of patients. Only 27.1% (482/1776) of patients were admitted to high-care or intensive care units. A decrease in respiratory virus detections was observed, except for RSV, EV and hCoV-OC43. RSV prevalence increased in 2021, while influenza A/B viruses remained undetected.

## 1. Introduction

The novel coronavirus that caused severe coronavirus disease in 2019 (COVID-19) was first detected in China in late December 2019 [1]. Since then, it was declared a Public Health Emergency and a pandemic, which has resulted in over 775,615,736 confirmed cases, including 7,051,323 deaths worldwide [2]. The first confirmed case of COVID-19 in South Africa was reported on 5 March 2020 [3]. 

The South African Government enforced a nationwide lockdown later that month, and several public health strategies were implemented in an effort to curb the plight of the pandemic [4]. COVID-19 is caused by the severe acute respiratory syndrome coronavirus 2 (SARS-CoV-2). SARS-CoV-2 is one of multiple respiratory viruses that significantly contribute to a large percentage of morbidity and mortality [5].

The other most common viruses involved in respiratory infections include adenovirus (AdV), enterovirus (EV), influenza A virus (Flu A), influenza B virus (Flu B), human bocavirus (hBoV), human metapneumovirus (hMPV), parainfluenza virus types 1 to 4 (PIV1-4), rhinovirus (RV), parechovirus (PeV), respiratory syncytial virus (RSV) and the four endemic human coronaviruses (hCoVs; -229E, -NL63, -OC43 and -HKU1) [6]. Pre-pandemic viral causative agents were estimated to be involved in up to 80% of respiratory illness cases, which range in severity [7,8].

Respiratory viruses are generally transmitted through droplet spread or direct contact with respiratory secretions [9]. The rate of transmission of respiratory viruses is multifactorial and affected by pharmaceutical and non-pharmaceutical interventions (NPIs) [10]. Literature indicates more than a 2-fold decrease in respiratory virus infections, from 49.8% during 2018–2019 to 39% in 2019–2020 and further to 13.4% during 2020–2021 [8]. This decline is partially attributed to the implementation of NPIs, which have reshaped the landscape of viral respiratory infections. These interventions include modifications in human behaviour, healthcare utilization, and the use of personal protective equipment amid the COVID-19 pandemic [8]. This situation challenges our comprehension of viral co-infection dynamics during the pandemic, particularly in the context of South Africa.

Detection of the causative agent in suspected viral respiratory infections is limited due to the cost–benefit ratio. The majority of viral respiratory infections are self-limiting and do not require intensive follow-up [11]. However, in the last decade, standard practices have been reformed with the familiarization and implementation of improved molecular diagnostic tools in the detection of respiratory pathogens [8]. The use of such diagnostic tools has assisted not only in clinical outcomes but also added tremendous value in respiratory pathogen surveillance and the development of prevention and treatment strategies [7].

Several studies have indicated an increase in the presence of co-existing respiratory viruses following the relaxation of NPI measures, with more cases occurring prior to the emergence of SARS-CoV-2 [12,13]. Additionally, other studies have associated this rise with geographical factors, aligning with established seasonal infection patterns. The pandemic has prompted advancements in scientific practices, notably in diagnostic tools and rapid testing, driven by heightened awareness of respiratory viral infections. However, local investigations into COVID-19 and concurrent respiratory virus infections, as well as the underlying reasons for fluctuations in co-circulating viruses, remain limited.

This study aimed to assess the prevalence as well as the seasonal patterns of respiratory virus infections during the SARS-CoV-2/COVID-19 pandemic among patients who accessed care at public health care facilities in Gauteng Province, South Africa, from 1 April 2020 to 30 March 2021.

## 2. Material and Methods

### 2.1. Study Setting and Population

This study was conducted in the Department of Virology, National Health Laboratory Service (NHLS) at Charlotte Maxeke Johannesburg Academic Hospital (CMJAH), Gauteng Province, South Africa. The study population included patients of all ages who attended public healthcare facilities in Gauteng Province, South Africa, for whom multiplex respiratory virus diagnosis, with or without SARS-CoV-2 diagnosis, was requested. Demographic and laboratory data were retrieved from the NHLS Corporate Data Warehouse (CDW) for the period from 1 April 2020 to 31 March 2021. Demographic data such as age, sex, specimen type, date of specimen collection, unique hospital number, ward assignment and health facility were retrieved. After the removal of duplicates, the data were anonymized through the removal of all patient identifiers like the unique hospital number.

### 2.2. Specimen Type and Total Nucleic Acid Extraction

Respiratory specimens received included nasopharyngeal swabs or oral swabs transported in a viral transport medium or sent as dry swabs, aspirates and sputum.

Total nucleic acid (TNA) extraction was required for all multiplex real-time PCR assays, except the BioFire Respiratory Panel 2.1 plus (RP2.1; BioFire Diagnostics, Salt Lake City, UT, USA) assay. TNA was extracted from 200 μL of respiratory specimens using the fully automated MagNA Pure 96 (MP96) extraction instrument coupled with the small volume TNA kit (Roche Diagnostics, Mannheim, Germany), as per the manufacturer’s instructions. TNA extraction was also performed on the micro lab NIMBUS automated extraction system using the STARMag Viral DNA/RNA kit (Seegene Inc., Seoul, Republic of Korea). For SARS-CoV-2 diagnosis, nucleic acid was also prepared as described [14].

### 2.3. Respiratory Virus Real-Time PCR Assays

Routine respiratory virus diagnosis was performed using the multiplex FastTrack Diagnosis (FTD) respiratory pathogens 21 polymerase chain reaction (PCR) test kit (Siemens Health engineers, Erlangen, Germany). The assay detects AdV, EV, Flu A and Flu B, hBoV, hMPV, PIV1 to 4, RV, PeV, hCoV-229E, hCoV-NL63, hCoV-OC43, hCoV-HKU1 and RSV. SARS-CoV-2 diagnosis was performed using the Allplex™ 2019-nCoV Assay (Seegene Inc., Seoul, Republic of Korea), the TaqPath COVID-19 assay (Thermo Fisher Scientific, Waltham, MA, USA), the Cobas^®^ SARS-CoV-2 assay (Roche Diagnostics, Mannheim, Germany) and the BioFire RP2.1 (Biofire Diagnostics, UT, USA) assay as described [15]. The BioFire RP2.1 assay detects the same respiratory viruses as the FTD assay as well as additional bacterial targets (*Bordetella pertussis*, *Bordetella parapertussis*, *Chlamydia pneumoniae* and *Mycoplasma pneumoniae*), which were excluded from the analysis.

## 3. Data and Statistical Analysis

The data retrieved was cleaned and categorized into Microsoft Excel (version 16) tables (Redmond, WA, USA). Incomplete or missing data were excluded from this study. Descriptive statistical analysis was conducted across different age groups, and cross-tabulations between categorical variables such as age groups, facility types and seasonality of respiratory viruses and SARS-CoV-2 results were summarized using proportions and percentages. The Z proportion test was used to compare the differences in coinfection rates between the 16 most common viral pathogens and SARS-CoV-2.

Disease severity was defined as patients with severe respiratory illness who were admitted to an intensive care unit (ICU) or high care unit (HCU) at the time of diagnosis. The association of these factors was assessed using the Chi-square test or Fisher’s exact test. To quantify the associations, the odds ratio was calculated together with its corresponding confidence intervals. A *p*-value of <0.05 represented statistical significance. All statistical analyses were performed using Stata statistical, version 17 (College Station, TX, USA).

## 4. Results

### 4.1. Patient Demographics

**Figure 1:**
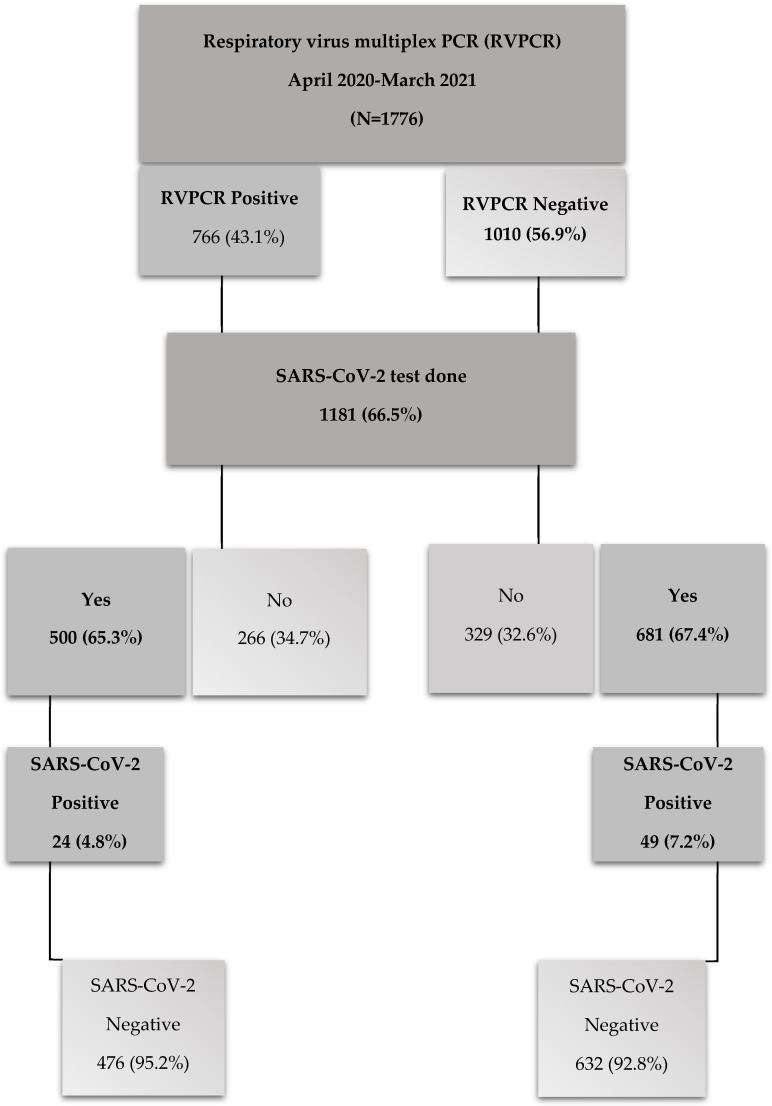
Flow chart describing the total number of patients tested for RSV, influenza and other respiratory viruses via respiratory multiplex PCR (RVPCR) test and the proportion of respiratory virus-positive and -negative cases that were also tested for SARS-CoV-2.

A total of 1776 patients for whom respiratory virus diagnosis was requested were included in this study, of which only 66.5% (1181/1776) were also tested for SARS-CoV-2 (Figure 1). The study population was dominated by young children between 0–2 years of age (1018/1776; 57.3%), 3–5 years (158/1776; 8.9%), 6–18 (182/1776; 10.2%), 19–64 (237/1776; 13.4%) and >65 years (181/1776; 10.2%). Males (870/1776; 49%) and females (853/1776; 48%) were equally represented in this study, with 3% (53/1776) for whom gender was not specified. The majority of patients were from general wards (1056/1776; 59.5%) and outpatient clinics (238/1776; 13.4%), with ICU accounting for 25.4% (451/1776) and HCU for only 1.7% (31/1776). Among patients who were categorized as having severe disease, a median age of 1.8 years (0–76 years) was reported.

### 4.2. Overall Prevalence of Respiratory Viruses

Among all respiratory samples, 43.1% (766/1776) tested positive for respiratory viruses other than SARS-CoV-2. RV (368/1776; 20.7%) was the most frequently detected respiratory virus, followed by RSV (304/1776; 17.1%), AdV (112/1776; 6.3%) and EV (105/1776; 5.9%). Among the four common coronaviruses, hCoV-OC43 (39/1776; 2.2%) was the most prevalent, with hCoV-229E, hCoV-NL63 and hCoV-HKU1 detected at frequencies of 0.7% (12/1776), 0.5% (9/1776) and 0.3% (6/1776), respectively. PIV4 (30/1776; 1.7%) was the most prevalent among the parainfluenza viruses, and hBoV was detected in 61/1776 (3.4%) respiratory samples.

Similar proportions of samples that tested positive (500/1181) and negative (681/1181) for other respiratory viruses were also tested for SARS-CoV-2. Co-detection of other respiratory viruses with SARS-CoV-2 was observed in 4.8% (24/500), with the most prevalent co-infections being RV, RSV and AdV. However, 7.2% (49/681) of samples were only SARS-CoV-2 positive (Figure 1). There was evidence that suggests those with PIV3 co-infection were at higher risk of having co-infection with SARS-CoV-2 (AOR: 40.91, 95% CI: 3.07–543.8) (*p* < 0.05). However, those with RSV (AOR: 0.31, 95% CI: 0.11–0.88) were shown to be statistically at a lower risk of co-infection (*p* < 0.05).

### 4.3. Age-Associated Prevalence of Respiratory Viruses

**Figure 2:**
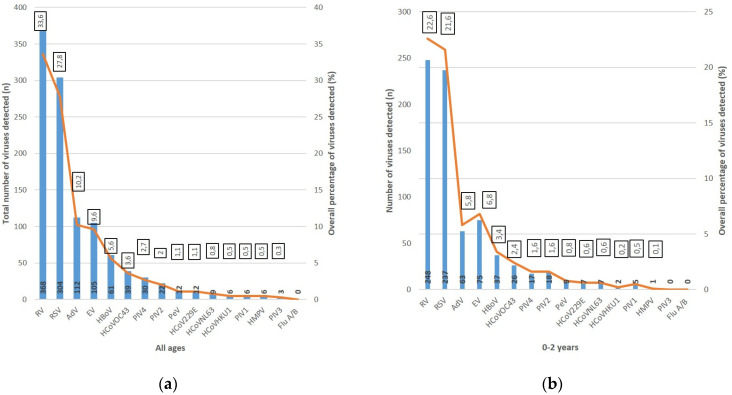

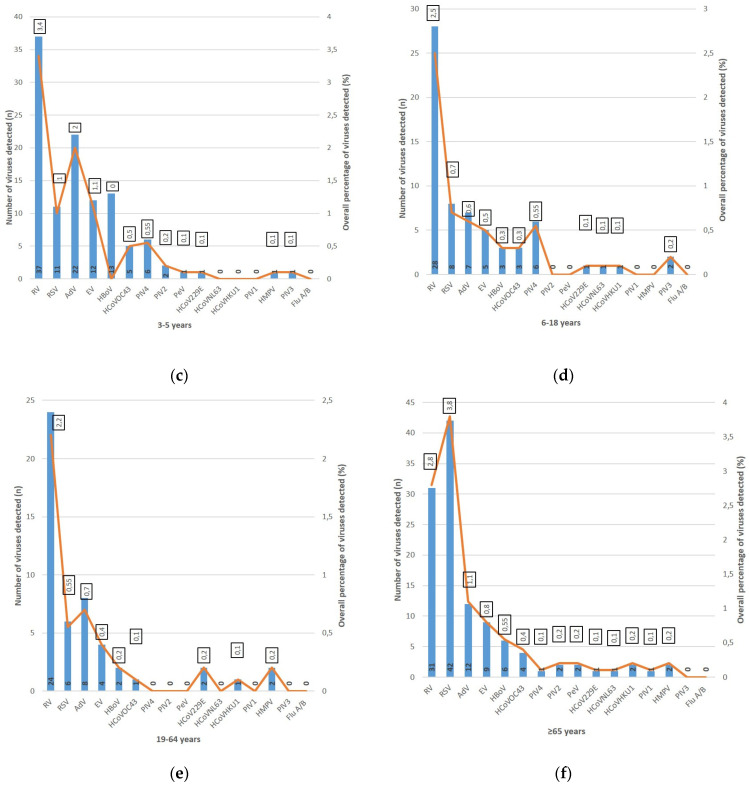
Total number and percentage of all respiratory viruses detected according to age from April 2020 to March 2021, Johannesburg, South Africa. (**a**) Total number and percentage of respiratory viruses detected in all ages. (**b**) Total number and percentage of respiratory viruses detected in 0–2 years. (**c**) Total number and percentage of respiratory viruses detected in 3–5 years. (**d**) Total number and percentage of respiratory viruses detected in 6–18 years. (**e**) Total number and percentage of respiratory viruses detected in 19–64 years. (**f**) Total number and percentage of respiratory viruses detected in >65 years.

Among the 766 RVPCR-positive patient samples, a total of 1095 respiratory virus detections were recorded. A total of 480/766 (62.7%) patients had a single virus infection, and 286/766 (37.3%) had multi-virus detections. RV (368/1095; 33.6%) was the most commonly detected pathogen, followed by RSV (304/1095; 27.8%) and AdV (112/1095; 10.2%). Among the patients with other respiratory virus infections, the majority (752/1095; 68.7%) were in the 0–2-year age group, followed by those >65-year old (121/1095; 11.1%) and 3–5-year old (112/1095; 10.2%) (Figure 2). RSV was most prevalent in the 0–2-year olds (237/304; 78%), followed by the over 65-year olds (42/304; 13.8%). RV and AdV were also more prevalent in this age group, with 67.4% (248/368) and 56.3% (63/112) detections, respectively.

### 4.4. Respiratory Virus Detections and Disease Outcomes

Notably, most patients infected with respiratory viruses experienced mild to moderate disease (838/1095; 76.5%). Overall, mild–moderate cases were primarily driven by RV (282/838; 33.7%) and RSV (217/838; 25.9%) infections. There were a total of 257/1095 severe cases (23.5%), of which only four cases (4/257; 1.6%) were co-infected with SARS-CoV-2 (Table 1). All four cases were in children < 6 months of age.

Among the total number of severe cases, RV (86/257; 33.5%) and RSV (87/257; 33.9%) were predominantly detected. In comparison, viruses associated with mild–moderate disease, where SARS-CoV-2 was also detected, included AdV (5/31; 16%), RSV (4/31; 13%), EV (2/31; 6%), hBoV (2/31; 6%), PIV3 and PIV4 (4/31; 13%) and other hCoVs (2/31; 6%) (Table 1). There was evidence that suggests that those with AdV had a lower risk (AOR: 0.51, 95% CI: 0.31–0.06) of developing severe disease compared to those without AdV (*p* < 0.05).

For hCoV-OC43, a total of 14/39 (35.9%) cases had a severe illness, followed by PIV4 with 10/30 (33.3%) cases, whereas 28.6% (87/304) of RSV infections had severe outcomes. hCoV-OC43 showed marginal statistical significance (AOR: 2.26, 95% CI: 0.97–5.27) with an increased risk of developing severe disease (*p* = 0.058). hBoV infections were the fifth most prevalent respiratory virus and were mainly seen in children ≤ 5 years, of which 18.0% (11/61) had a severe illness. Those who tested positive for RVPCR and who were classified as having severe disease were seen predominantly in the age groups ≤ 5 years (151/257; 58.8%). Viruses that were associated with severe disease in this age category included (mean age; SD) PIV4 (0.3; 0.43), hCoV-OC43 (0.9; 1.06), EV (1.2; 2.2), RSV (1.3; 5.5), RV (1.9; 4.5), hBoV (2.1; 3.2) and AdV (4.4; 7.5).

### 4.5. Seasonal Distribution of Respiratory Viruses

**Figure 3:**
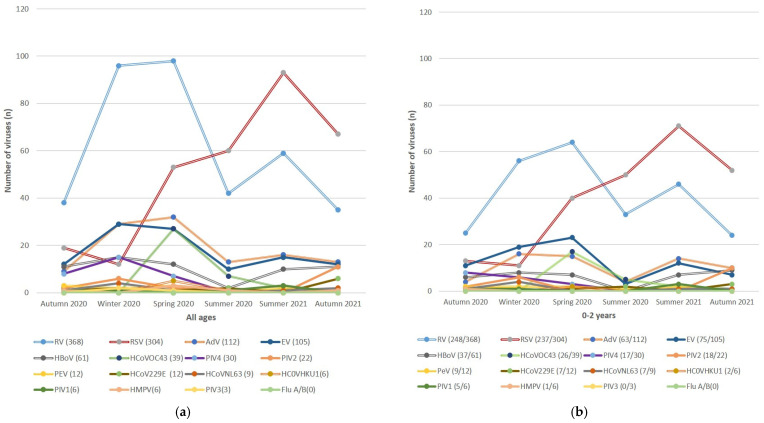

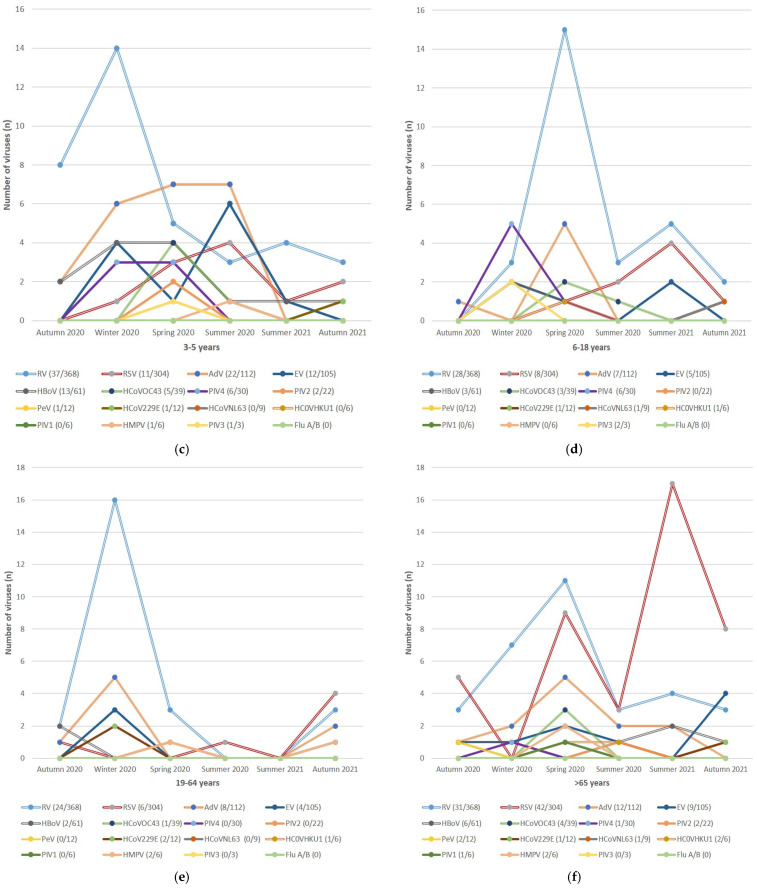
Pattern of the seasonality of respiratory viruses from April 2020 to March 2021 in Johannesburg, South Africa. (**a**) Pattern of the seasonality of respiratory viruses in all ages. (**b**) Pattern of the seasonality of respiratory viruses in 0–2 years. (**c**) Pattern of the seasonality of respiratory viruses in 3–5 years. (**d**) Pattern of the seasonality of respiratory viruses in 6–18 years. (**e**) Pattern of the seasonality of respiratory viruses in 19–64 years. (**f**) Pattern of the seasonality of respiratory viruses in >65 years.

The pattern of seasonality for respiratory viruses (excluding SARS-CoV-2) from 1 April 2020 to 31 March 2021 is shown in Figure 3. AdV was consistently detected throughout the winter of 2020 (29/112; 25.9%), the spring of 2020 (32/112; 28.6%) and the summer of 2020/2021 (29/112; 25.9%). Similar patterns of circulation were noted for RV, with higher periods of detection during the winter of 2020 (94/368; 25.5%), spring of 2020 (98/368; 26.6%) and summer of 2020/21 (101/368; 27.4%). RSV was detected at lower levels compared to RV during the autumn and winter of 2020 (31/304; 10.2%), with peak detection in the summer of 2020/21 (153/304; 50.3%), exceeding RV detections.

EV detections peaked during the winter of 2020 (29/105; 27.6%) and the spring of 2020/21 (27/105; 25.7%). Thereafter, lower levels of EV were detected in the summer of 2020/2021 (25/105; 23.8%) and autumn 2021 (12/105; 11.4%). PIV4 was the most prevalent PIV, with a peak (15/30; 50%) observed in the winter of 2020. Peak detection of PIV2 was observed in the autumn of 2021 (11/22; 50%) compared to a lower peak in the winter of 2020 (6/22; 27.3%). No Flu A or Flu B viruses were detected during the study period.

hCoV-OC43 detections peaked during the spring of 2020 (27/39; 69.2%). Higher levels of hCoV-229e (3/12; 25%) and hCoV-NL63 (5/9; 55.5%) were mainly detected in autumn and winter compared to hCoV-HKU1, which was mainly detected in the spring (5/6; 83.3%).

## 5. Discussion

This study aimed to determine the prevalence and describe the seasonal patterns of respiratory viruses during the COVID-19 pandemic. During that period, NPIs were implemented to limit the spread of SARS-CoV-2, reducing the stress placed on the public healthcare system and the economy in South Africa [16]. In this study, we reviewed the laboratory data on the prevalence of respiratory viruses detected among patients who used public healthcare facilities, spanning all age groups. There is now substantial global evidence that alludes to changes in the previously described prevalence and seasonality of respiratory viruses attributable to COVID-19 [17]. The overall prevalence of circulating respiratory viruses was higher for RV and RSV. Our findings were unsurprising, considering that our study population was predominately weighted by children under 5 years of age and their association with RV and RSV as leading causes of respiratory infection in children [18]. However, the majority of other non-enveloped respiratory viruses, i.e., AdV, hBoV and EV, were more prevalent than other enveloped viruses, like hCoVs and PIV. This variation may be attributable to the viral structure of non-enveloped viruses rather than their seasonal patterns [18]. However, further research is needed to reach definitive conclusions.

A significant change was observed in the typical seasonal patterns of respiratory virus infections within our setting (Figure 3). South Africa has a well-defined seasonal pattern for viruses such as influenza and RSV. The RSV seasonality typically precedes the influenza season, beginning in February and continuing through mid-May [19]. However, this study found no circulating Flu A or B viruses as expected during the winter months. Lower Flu A and B virus circulation was also reported in the United States (US), with RSV positivity dropping from 15.3% to 1.4% in 2020 and remaining at <1% for the next year [19]. Furthermore, our findings were similar to Madagascar, where the circulation of influenza viruses was not seen from March 2020 until late July 2021. This is consistent with the sporadic circulation of Flu A and B viruses and the lower prevalence of RSV previously reported at the national level in South Africa during the 2020 SARS-CoV-2 pandemic [20,21].

Similar findings were noted for RV and AdV, with prevalences of 33.6% and 10.2%, respectively. The year-round detection of RV and AdV correlated with previous local studies albeit at lower levels compared to pre-pandemic levels [22]. RV/EV was the most prevalent respiratory virus other than SARS-CoV-2, despite a drop in overall prevalence as observed for all other respiratory viruses in the US.

Multiple factors could be considered in determining why there was a shift in seasonal patterns during the pandemic. Firstly, lockdown restrictions, which included cancellation of social gatherings, international and local border closures and movement restrictions and then eased to social distancing, continuation of face mask use, and hand hygiene [23], dramatically changed transmission routes. These measures contributed to the low levels of virus circulation during the 2020 season. However, not all viruses were equally susceptible to lockdown measures, as seen by the relatively higher number of cases of AdV and RV in 2020 [24].

In comparison, the impact of COVID-19 control measures on the spread of influenza and RSV was greater and may be related to their effective reproductive numbers (R0) [25]. The R0 of seasonal influenza (1.2–1.4) is estimated to be lower than that of RSV (1.7–2.1), suggesting that the COVID-19 control measures had a more significant impact on influenza and its transmission [20]. There was a sudden resurgence of RSV infections during the summer and autumn of 2021 compared to the year-round infection rates of RSV in 2020. This sudden increase may be attributed to exposure and transmission within a susceptible population, heightened by the reopening of schools [26].

In this study, PIVs displayed year-round circulation, with PIV4 being the most prevalent, with 50% of cases detected in the winter of 2020, followed by PIV2, with a lower detection rate (27.3%) during the same period but a higher peak prevalence (59.1%) in the autumn of 2021. Similarly, for PIV1, PIV2 and PIV3, it was observed that over a 6-year period (2009–2014), year-round circulation occurred in South Africa [27]. Parsons et al. (2023) also reported dominance of PIV4 and PIV3, with peaks in autumn/winter and late winter/spring, respectively, in the Western Cape of South Africa from 2014 to 2019. However, following the COVID-19 pandemic, they observed a sustained drop in the prevalence of PIV4 compared to PIV3 after containment measures were lifted [28]. Unfortunately, they had no PIV-type-specific data for the period under investigation in this study, but the dominant PIV type in late 2020 to May 2021 was PIV3, with PIV4 peaking at a lower level from March to July 2021 (autumn–winter) and continuing to circulate at low levels thereafter. However, like their findings, we observed peak detection of PIV4 in the winter of 2020, where after detections dropped to low levels/sporadic detections in the autumn of 2021, whereas PIV2 peaked during this period. The seasonality is similar to that described in other parts of the world and underscores the resistance to NPI measures [29].

hCoVs other than SARS-CoV-2 were detected throughout the study period but at lower levels compared to pre-pandemic rates [29]. In this study, we reported that among the four common coronaviruses, hCoV-OC43 (2.2%) was the most frequently detected (albeit at a lower prevalence), which contrasts with a study conducted in 2012–2013 that reported hCoV-229E (4.1%) as the most prevalent hCoVs [29]. Madagascar also reported a dominant circulation of hCoV-OC43 during the spring/summer of 2020 at an overall prevalence of 3.7%, which was higher than in South Africa [21]. Similar to our observations, the circulation of hCoV-OC43 dropped to low levels thereafter. The seasonal distribution of common hCoVs is not well described in South Africa and was not discussed by Subramoney et al. (2018) [29]. In the same study, hBoV was detected at a prevalence of 3.7% (25/680), which is similar to our finding of a prevalence of 3.4% in 2020. In the US, hCoV positivity also dropped from 7% and remained at <1% in the first year of the pandemic, and when it rebounded in 2021, hCoV-OC43 and hCoV-NL63 were mainly detected [19]. This is similar to local studies, which have shown continued detection at a lower rate after the lockdown period [22].

Overall prevalence rates of respiratory viruses varied across different age groups, with the most frequently detected respiratory viruses being RV, RSV, AdV and EV. These results may have a significant bias, as our study population was heavily weighted within the paediatric population. However, we included age-specific categories to limit this bias, albeit at lower rates. Among other local studies, the pooled prevalence rates of RSV varied between 17 and 25% across all age groups, predominantly higher in those ≤5 years old [30]. In this study, we report a similar overall prevalence rate of 27.8%, with an increased prevalence in the age category ≤ 5 years. In keeping with other studies, most infections were noted in extremes of age (≤5 and >65 years) [31]. The same can be noted for RV and AdV, where our results indicate that the age group ≤ 5 years is at a higher risk of infection. These findings suggest that there has been no significant change in age-related risk, and it is most likely due to immunosenescence at the extremes of age.

These age groups were also noted to be at high risk for severe disease and further complications. Notably, most patients infected with respiratory viruses experienced mild to moderate disease (76.5%). Overall, mild–moderate and severe cases were primarily driven by RV and RSV infections. Several severe cases in this study had no evidence of respiratory infection, either COVID-19 or other respiratory viruses, as they tested negative for RVPCR and SARS-CoV-2. This suggests that severe cases could be due to other pathogens or disease entities. However, though there were no signs of respiratory infection at the time, one must take into consideration that testing and detection of respiratory pathogens could have been delayed or missed. The reasoning is multifactorial, and the total testing process must be considered. Establishment and changes in viral dynamics at the time, improper collection technique, site of collection and false negative results are points to consider. Similar studies suggest that underlying respiratory disease, diabetes mellitus, tuberculosis, HIV, COVID-19 vaccination status and obesity (BMI ≥ 30) are associated with high risk factors for disease progression [32]. Unfortunately, our study did not include these parameters, and no associations could be made.

There was a total of 24 cases co-infected with SARS-CoV-2. RV, RSV and AdV were again the most commonly detected co-infected viruses in this group. Co-infection with at least one other respiratory virus was more common among RV and RSV-positive cases. The predominance of RV and RSV in both single and multiple detections is in agreement with previously published data [33]. Our study findings show that SARS-CoV-2 co-infection with other respiratory viruses was low, and there was a higher incidence of SARS-CoV-2 mono-infection. Theories include viral and host factors that limit disease progression. SARS-CoV-2 has been documented to limit the replication of other respiratory viruses in a term referred to as viral interference [34]. Although co-infection with two or more viruses is not uncommon, competition between viruses limits the co-infection rate and, at times, disease progression [35]. Our subgroup analyses revealed that the rate of co-infection was significantly higher in the paediatric population compared to the adult group, as well as a higher incidence of COVID-19 disease without co-infection. These results are consistent with a recent study examining co-infection rates in paediatric and adult patients [36]. Of the total positive RVPCR results, there was a 65.3% dual test rate with SARS-CoV-2. This may limit our ability to safely draw conclusions on SARS-CoV-2 co-infections within our study population, which was <5%. However, other studies show that children < 2 years of age had the highest frequency of co-infection and detection of any pathogen, including SARS-CoV-2 [37]. Our study findings align with this pattern, with the majority of detectable pathogens falling within this age group. It is possible that if our cohort age increased, we would see an even lower co-infection rate, as one study showed that with every 1-year increase in age, the rate of co-infections decreased by 8% [38].

This suggests that co-infections were limited. Factors associated with this decrease may include host age, time of infection, distinct viral characteristics and differences in their transmission dynamics [39]. Additionally, there may already be a degree of immunity to common respiratory viruses within a population. It is possible that a higher viral inoculum is needed for effective transmission and subsequent disease progression in these groups.

The above findings highlight the potential effects that the introduction of RSV and other pipeline vaccines may have. Overall, there were notable changes in respiratory virus circulation, especially for RSV and influenza. Our data show low-level circulation of other viruses (AdV, RV, hCoV-OC43, EV, PIV4 and 2 and hBoV) across all age groups but predominantly in the younger population of 0–5 years. Global studies have shared similar findings to ours; a retrospective study in the US reported a persistence of RV and EV detection, with similar patterns observed in the Asian-pacific region, including fluctuating levels of AdV and hBoV [13,40]. This suggests that, in the absence of high levels of RSV and influenza, these viruses will predominate in circulation at varying levels across different age groups.

Statistical models predict a long period (6 years) for compound vaccine effects [40]. Not only will there be a change in seasonal trends of viruses, but it may also result in a delayed onset of primary infection and changes in disease severity. Our study showed that the largest number of RSV infections outside of the 0–5 years age group was >65 years. This suggests a higher likelihood of poor disease outcomes in this age group if more individuals are infected later in life. However, the inverse can be said about non-enveloped viruses, which are associated with milder disease severity and hospitalizations. This will reduce the stress placed on the healthcare sector, which will have the greatest impact on lower-middle-income regions such as South Africa. The sudden drop in respiratory virus transmission may affect the genetic diversity of many viral species and will create a long-lasting effect [13]. However, continued surveillance and monitoring will be required to truly understand changes in viral respiratory evolution and their potential downstream effects.

There are several limitations in this study that should be considered. Firstly, this is a single-centered study conducted over a one-year period. This limits us in comparing all seasons and populations. Secondly, the testing methods for SARS-CoV-2 changed over time to adapt to the demands of the pandemic. However, all assays implemented were verified, and the diagnosis was based on manufacturers’ specifications and global interpretation guidelines. However, this could have led to variations in sensitivity and specificity between tests, possibly resulting in missed detections of respiratory viruses and, subsequently, fewer co-infections being identified. Thirdly, our cohort was predominately less than 5 years of age, which may not accurately represent our general population and may skew our findings with regard to detectable viral pathogens, co-infection rate, seasonality and disease severity. Lastly, more clinical data could have been elicited to use as clinical endpoints to determine disease severity.

## 6. Conclusions

Although a change in the seasonal patterns of respiratory viruses was seen in Gauteng Province, South Africa, during the COVID-19 pandemic, most respiratory viruses, with the exception of Flu A and B viruses, were detected, albeit at low levels. This highlights the effectiveness of NPIs during the study period. Understanding respiratory virus circulation, distribution and seasonal patterns is required for informed decision-making on the implementation of public health disease prevention and management strategies. Notably, from this study, it is clear that data on the seasonality of respiratory viruses other than Flu A and B viruses and RSV are still lacking in South Africa and Africa. Although co-infection with two or more viruses is not uncommon, competition between viruses was observed, as co-infections of SARS-CoV-2 with other respiratory viruses were infrequent, suggesting that SARS-CoV-2 infection did not increase the likelihood of co-infections with other respiratory viruses. Furthermore, this implies that continued monitoring of local epidemiology in the coming years is important. This will help to assess whether the effects on the circulation of other respiratory viruses during the pandemic are permanent or transient. During outbreaks, it is crucial to understand fluctuations within the respiratory viral landscape and the effectiveness of NPIs to improve ongoing management practices.

## Figures and Tables

**Table 1:** Percentage and frequency of respiratory viruses detected via multiplex PCR according to age, severity and co-infection with SARS-CoV-2 in Johannesburg, South Africa.

VIRUS	SARS-CoV-2 POSITIVE n (%)		AGE n (%)					Severity n (%)	
			0–2	3–5	6–18	19–64	65+	Mild-Moderate	Severe
**RV** **(368)**	12 (3.2%)	Severe: 4	248 (67.4%)	37 (10.1%)	28 (7.6%)	24 (6.5%)	31 (8.4%)	282 (76.6%)	86 (23.4%)
		Moderate: 8							
**RSV** **(304)**	4 (1.3%)	Severe: 0	237 (78%)	11 (3.6%)	8 (2.6%)	6 (2%)	42 (13.8%)	217 (71.4%)	87 (28.61%)
		Moderate: 4							
**AdV** **(112)**	5 (4.5%)	Severe: 0	63 (56.3%)	22 (19%)	7 (6.3%)	8 (7.1%)	12 (10%)	94 (83.9%)	18 (16.1%)
		Moderate: 5							
**EV** **(105)**	2 (1.9%)	Severe: 0	75 (71.4%)	12 (11.4%)	5 (4.8%)	4 (3.8%)	9 (8.6%)	84 (80%)	21 (20%)
		Moderate: 2							
**hBoV** **(61)**	2 (3.3%)	Severe: 0	37 (60.6%)	13 (21.3%)	3 (4.9%)	2 (3.3%)	6 (9.8%)	50 (82%)	11 (18%)
		Moderate: 2							
**hCoV-OC43** **(39)**	0 (0%)		26 (66.7%)	5 (12.8%)	3 (7.7%)	1 (2.7%)	4 (10.2%)	25 (64.1%)	14 (35.9%)
**PIV4** **(30)**	2 (6.7%)	Severe: 0	17 (56.7%)	6 (20%)	6 (20%)	0 (0%)	1 (1%)	20 (66.7%)	10 (33.3%)
		Moderate:2							
**PIV2** **(22)**	0 (0%)		18 (81.8%)	2 (9.1%)	0 (0%)	0 (0%)	2 (9.1%)	18 (81.8%)	4 (18.2%)
**PeV** **(12)**	0 (0%)		9 (75%)	1 (8.3%)	0 (0%)	0 (0%)	2 (16.7%)	11 (91.7%)	1 (8.3%)
**hCoV-229E** **(12)**	1 (8.3%)	Severe: 0	7 (58.3%)	1 (8.3%)	1 (8.3%)	2 (16.7%)	1 (8.3%)	10 (83.3%)	2 (17%)
		Moderate: 1							
**hCoV-NL63** **(9)**	1 (11.1%)	Severe: 0	7 (77.8%)	0 (0%)	1 (11.1%)	0 (0%)	1 (11.1%)	8 (88.9%)	1 (11.1%)
		Moderate: 1							
**hCoV-HKU1 (6)**	0 (0%)		2 (33.3%)	0 (0%)	1 (16.7%)	1 (16.7%)	2 (33.3%)	5 (83.3%)	1 (16.7%)
**PIV1** **(6)**	0 (0%)		5 (83.3%)	0 (0%)	0 (0%)	0 (0%)	1 (16.7%)	5 (83.3%)	1 (16.7%)
**hMPV** **(6)**	0 (0%)		1 (16.7%)	1 (16.7%)	0 (0%)	2 (33.3%)	2 (33.3%)	6 (100%)	0 (0%)
**PIV3** **(3)**	2 (66.7%)	Severe: 0	0 (0%)	1 (33.3%)	2 (66.7%)	0 (0%)	0 (0%)	3 (100%)	0 (0%)
		Moderate: 2							
**Flu A/B** **(0)**	0 (0%)		0 (0%)	0 (0%)	0 (0%)	0 (0%)	0 (0%)	0 (0%)	0 (0%)
**TOTAL** **1095**	31 (2.8%)	Severe: 4	752 (68.7%)	112 (10.2%)	65 (5.9%)	50 (4.6%)	116 (10.6%)	838 (76.5%)	257 (23.5%)
		Moderate: 27							

## Data Availability

The raw data were generated by the National Health Laboratory Service’s Corporate Data Warehouse. Derived data supporting the findings of this study are available from the corresponding author, B.R., upon suitable and fair request.
